# Depletion of *m*-type thioredoxin impairs photosynthesis, carbon fixation, and oxidative stress in cyanobacteria

**DOI:** 10.1093/plphys/kiab321

**Published:** 2021-07-15

**Authors:** Manuel J Mallén-Ponce, María José Huertas, Ana María Sánchez-Riego, Francisco J Florencio

**Affiliations:** Instituto de Bioquímica Vegetal y Fotosíntesis, Universidad de Sevilla-CSIC, Américo Vespucio 49, 41092 Sevilla, Spain

## Abstract

Thioredoxins (Trxs) are disulfide oxidoreductases that regulate many biological processes. The *m*-type thioredoxin (TrxA) is the only Trx present in all oxygenic photosynthetic organisms. Extensive biochemical and proteomic analyses have identified many TrxA target proteins in different photosynthetic organisms. However, the precise function of this essential protein in vivo is still poorly known. In this study, we generated a conditional *Synechocystis* sp. PCC 6803 mutant strain (STXA2) using an on-off promoter that is able to survive with only 2% of the TrxA level of the wild-type (WT) strain. STXA2 characterization revealed that TrxA depletion results in growth arrest and pronounced impairment of photosynthesis and the Calvin–Benson–Bassham (CBB) cycle. Analysis of the in vivo redox state of the bifunctional enzyme fructose-1,6-bisphosphatase/sedoheptulose-1,7-bisphosphatase showed higher levels of oxidation that affected enzyme activity in STXA2. This result implies that TrxA-mediated redox regulation of the CBB cycle is conserved in both cyanobacteria and chloroplasts, although the targets have different evolutionary origins. The STXA2 strain also accumulated more reactive oxygen species and was more sensitive to oxidative stress than the WT. Analysis of the in vivo redox state of 2-Cys peroxiredoxin revealed full oxidation, corresponding with TrxA depletion. Overall, these results indicate that depletion of TrxA in STXA2 greatly alters the cellular redox state, interfering with essential processes such as photosynthetic machinery operativity, carbon assimilation, and oxidative stress response. The TrxA regulatory role appears to be conserved along the evolution of oxygenic photosynthetic organisms.

## Teaser

A cyanobacterial mutant strain with very low TrxA (*m*-type thioredoxin) content is unable to maintain cellular redox state, affecting the function of essential processes.

## Introduction

Thioredoxins (Trxs) belong to a large multigenic family of oxidoreductases found in all free-living organisms (Holmgren, 1985; Schürmann and Buchanan, 2008). These small soluble proteins share a characteristic fold, a conserved active site WC(G/P)PC sequence and a low redox potential that confers on them strong reductive properties. The two cysteines of the active site are involved in thiol/disulfide exchanges and can regulate a large number of cellular processes through the reduction of specific disulfide bridges of their target proteins (Holmgren et al., 2005). These Trxs are very abundant and diverse among oxygenic photosynthetic organisms, unlike other groups. In cyanobacteria, the availability of genome sequences showed the existence of four types, three of them corresponding to *m* (TrxA), *x* (TrxB), and *y* (TrxQ) types present in plant chloroplast, while the fourth thioredoxin, TrxC, is unique to cyanobacteria (Florencio et al., 2006). While the genome of *Synechocystis* sp. PCC 6803 (hereafter *Synechocystis*) contains only one copy of each of these Trxs, other cyanobacteria including *Anabaena* spp. contain up to three isoforms of TrxA namely Trx-*m*1, Trx-*m*2, and Trx-*m*3, in addition to TrxB, TrxC, and TrxQ (Florencio et al., 2006). In both *Synechocystis* and *Anabaena* sp. PCC 7120, quantitative immunoblotting indicated that TrxA (Trx-*m*1) is the most abundant Trx (Florencio et al., 2006; Mihara et al., 2020). In the case of land plants, using the plant model Arabidopsis (*Arabidopsis thaliana*) as reference, up to 20 different Trx isoforms are found and classified into seven groups: *m*1-4, *f*1-2, *x*, *z*, and *y*1-2 located in the plastid, *o*1-2 located in mitochondria and nucleus, and *h*1-8 distributed between cytosol, nucleus, endoplasmatic reticulum, and mitochondria (Geigenberger et al., 2017). These Trxs are mainly reduced by thioredoxin reductases: (1) ferredoxin–thioredoxin reductases (FTRs), which use ferredoxin as electron donor. FTRs are heterodimeric enzymes composed of a catalytic subunit containing an Fe-S group (FTRC) and a variable subunit (FTRV). FTRs are present in chloroplasts and in most cyanobacteria such as *Synechocystis* or *Anabaena* sp. PCC 7120 (Balsera et al., 2014). (2) Flavin thioredoxin reductases that are a heterogeneous group of enzymes including NAD(P)H-thioredoxin reductases (NTRs). NTRs accept reducing equivalents from NADPH and are highly conserved among all organisms. *Synechocystis* lacks this protein previously known as NTR (*slr0600*) that has recently been shown not to use or bind NADPH or NADH, is unable to reduce any thioredoxin and has been renamed as diflavin-linked disulfide oxidoreductase (DDOR). DDOR possibly functions as an oxidase (Buey et al., 2017a). Other members of this group are the ferredoxin flavin-thioredoxin reductases, which coexist with the FTRs in a reduced number of cyanobacteria, including the marine *Prochlorococcus* spp. group or the ancient cyanobacterium *Gloeobacter* spp (Buey et al., 2017b, 2021). Finally, chloroplasts and some cyanobacteria also present an NADPH-thioredoxin reductase (NTRC), containing both an NTR and a Trx domain in a single polypeptide (Serrato et al., 2004). NTRC appears to act as the primary reducing system for the 2-Cys peroxiredoxin (2-Cys Prx) (Pérez-Ruiz et al., 2006; Pascual et al., 2011). In *Synechocystis*, which lacks NTRC, its 2-Cys-Prx is reduced by Trxs via FTR (Pérez-Pérez et al., 2009b).

In oxygenic photosynthetic organisms, several enzymes of the Calvin–Benson–Bassham (CBB) cycle are redox regulated in a light-dependent manner, with thioredoxins playing a crucial role in this process (Schürmann and Buchanan, 2008). In cyanobacteria, the early studies revealed the capacity of Trxs to activate cyanobacterial fructose-1,6-bisphosphatase/sedoheptulose-1,7-bisphosphatase (FBP/SBPase; Schmidt, 1981; Crawford et al., 1984; Ip et al., 1984; Gerbling et al., 1986) and also by the light/dark cycles (Pelroy et al., 1976; Crawford et al., 1984). Other CBB enzymes as phosphoribulokinase (PRK) and CP12 also been found to be regulated by light/dark cycles in different organisms. Recently, the structures of the GAPDH/CP12/PRK complex and PRK from *Thermosynechococcus elongatus* BP-1 and *Synechococcus elongatus* PCC 7942 were solved and revealed the regulations of redox signaling for the PRK and CP12 in cyanobacteria (McFarlane et al., 2019; Wilson et al., 2019; Yu et al., 2020).

CBB cycle enzymes, such as FBPase and SBPase, are not evolutionarily related between cyanobacteria and chloroplasts (Jiang et al., 2012). In fact, cyanobacterial, FBP/SBPase activities resides in the same protein (Gerbling et al., 1986) and is essential for photoautotrophic growth (Tamoi et al., 1999; Yan and Xu, 2008). In plants, each enzyme activity corresponds to a different protein (Schürmann and Buchanan, 1975). Recently, structural and biochemical characterization of FBP/SBPase has suggested a possible redox-dependent activity in cyanobacteria (Ansong et al., 2014; Feng et al., 2014).

On the other hand, proteomic studies have also shown putative target proteins of TrxA, TrxB, and TrxQ related to cellular processes such as oxidative stress response in cyanobacteria (Lindahl and Florencio, 2003; Pérez-Pérez et al., 2006; Mata-Cabana et al., 2007; Nomata et al., 2015). In *Synechocystis*, these studies reported a high overlap on the target proteomes of TrxA, TrxB, and TrxQ, although this did not necessarily reflect the in vivo role of each Trx (Florencio et al., 2006). The photosynthetic electron transport chain is considered the main source of reactive oxygen species (ROS) in all photosynthetic organisms, including cyanobacteria (Apel and Hirt, 2004; Latifi et al., 2009). For ROS scavenging, these organisms have different antioxidant mechanisms (Latifi et al., 2009; Pathak et al., 2019). In *Synechocystis*, in vitro analysis of protein interactions and enzymatic activities, showed that peroxiredoxins (Prxs), which constitute a class of thiol-dependent peroxidases, can interact with and receive electrons from the different Trxs (Pérez-Pérez et al., 2009b).

The analysis of the in vivo role of the different Trxs has been made possible by the generation of mutant strains. In *Synechocystis*, using mutant strains lacking TrxB, TrxQ, and TrxC, it was shown that TrxB and TrxQ play a role in the oxidative stress response, including sensitivity to high light, while TrxC influences adaptation to low carbon conditions (Pérez-Pérez et al., 2009a; López-Maury et al., 2018). In contrast, it was not possible to obtain a mutant strain lacking TrxA, suggesting an essential role for this Trx (Navarro and Florencio, 1996). In fact, mutants lacking *m*-type Trx are not available up to now in oxygenic photosynthetic organisms. In the nitrogen-fixing cyanobacterium *Anabaena* sp*.* PCC 7120 only single mutants lacking Trx-*m*1 or Trx-*m*2 isoforms were obtained. These mutants had a similar growth-rate to the wild-type (WT) under standard growth conditions, suggesting that different isoforms can compensate for each other (Deschoenmaeker et al., 2018; Mihara et al., 2018; Deschoenmaeker et al., 2019). Interestingly, Trx-*m*1 mutant is unable to grow in nitrogen-fixing conditions (Mihara et al., 2018). In Arabidopsis, inactivation of all four *m*-types genes has also not been achieved; only mutants with single or combined deficiencies in the Trx*-m*1, Trx*-m*2, and Trx*-m*4 have been described, revealing specific functions of the different Trx-*m* isoforms (Courteille et al., 2013; Wang et al., 2013; Okegawa and Motohashi, 2015; Thormählen et al., 2017; Okegawa and Motohashi, 2020).

Even though the in vitro functions of the TrxA have been analyzed, nothing is known about its in vivo role in cyanobacteria. In this study, we generated and analyzed a conditional *Synechocystis* strain mutant where the *trxA* gene is transcribed under a regulated promoter that allows us to control the amount of TrxA. We show how TrxA depletion below 10% of the WT strain severely affects photosynthetic metabolism and redox balance in cyanobacteria.

## Results

### Drastic decrease of thioredoxin A in STXA2 severely affects its growth

**Figure 1.**
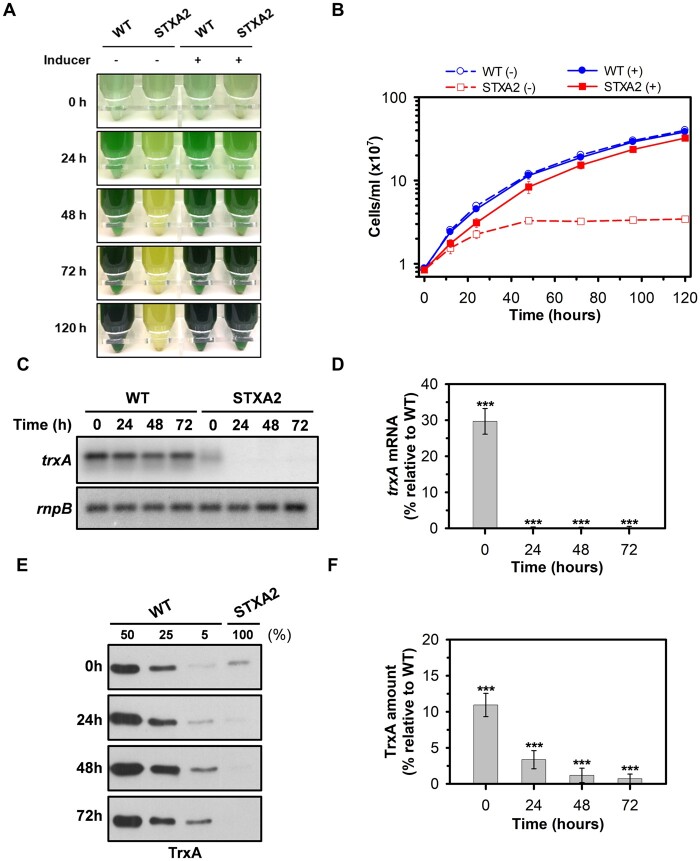
TrxA-dependent growth of STXA2 mutant. WT and STXA2 strains were grown in BG11C containing arsenite (1 mM) and transferred to medium with or without inducer. A, Photographs and (B) growth curve of WT and STXA2 strains in the presence or the absence of inducer at different times. C, Northern blot of *trxA* gene before (0 h) and after inducer removal (24–72 h). Total RNA was isolated from WT and STXA2 cells at different times. Five micrograms were analyzed and gel was blotted and hybridized with *trxA* probe. The filter was stripped and rehybridized with an *rnpB* probe as a loading control. D, Quantification of the relative mRNA levels of *trxA* in the STXA2 strain relative to the WT. Radioactive signals were quantified and normalized to the *rnpB* signal using ImageJ software. E, Western blot analysis of TrxA in the WT and STXA2 strains before (0 h) and after inducer removal (24–72 h). From the same cultures used for Northern blot analysis, samples equivalents to 4 ×10^7^ cells were taken at the indicated times, and total proteins were isolated and resolved on SDS–PAGE, blotted, and incubated with anti-TrxA antibody. Different dilutions of total protein extracts from WT cells were loaded (50%, 25%, and 5%) to calculate TrxA protein levels in the STXA2 strain. F, Quantification of TrxA protein levels in the STXA2 strain relative to the WT from results of (E) using ImageJ software. Data are means ± sd from three biological replicates in all cases. ***P <* *0.001 (two-tailed Student’s *t* test between WT and STXA2 strains).

Multiple sequence alignments of cyanobacterial TrxA sequences indicated a high degree of sequence identity to each other (>80%; Florencio et al., 2006). Even more, TrxA (*m*-type) is highly conserved compared to plants *m*-type Trx, showing 77%, 83%, and 85% protein sequence similarity to the C-terminal regions of Arabidopsis Trx-*m*1, Trx-*m*2, and Trx-*m*4, respectively. In cyanobacteria, several studies have shown that TrxA is an essential protein in cyanobacteria *Synechocystis* sp. PCC 6803 and *Synechococcus* sp. PCC 6301, but specific roles have not been associated with this Trx (Muller and Buchanan, 1989; Navarro and Florencio, 1996). To address this question, a conditional knockdown mutant was generated in *Synechocystis* containing the *trxA* gene under the control of the *arsB* promoter, which responds to the presence of arsenite in the medium (López-Maury et al., 2003), and a spectinomycin/streptomycin resistance cassette was inserted in the *trxA* original locus (Supplemental Figure S1A). The complete segregation of the new strain named STXA2 was verified by PCR analysis (Supplemental Figure S1B). Then we checked whether the growth of STXA2 is affected by its TrxA levels in standard growth conditions. For this purpose, WT and STXA2 strains were cultivated in media containing 1 mM of sodium arsenite (henceforth referred to as inducer), which is the concentration used for segregation of the original *trxA* locus, and transferred to new media with or without inducer after cell centrifugation (Figure 1, A and B). In the presence of inducer, both strains show a similar growth curve with slight growth retardation in the STXA2 strain (Figure 1B). The absence of the inducer caused a growth impaired phenotype in STXA2 and its complete growth arrest within 48–72 h (Figure 1, A and B). Northern and western blot were performed on selected points along the growth curve. The *trxA* expression is not affected by inducer removal in the WT, whereas the amount of *trxA* decreased to undetectable levels in STXA2 relative to the WT (Figure 1, C and D). Western blot analysis showed that TrxA protein level was about 10% in STXA2 of that in the WT in the presence of inducer (Figure 1, E and F). TrxA levels decreased progressively in STXA2 after inducer removal and reached <2% of that in the WT at 48 h (Figure 1, E and F).

**Figure 2:**
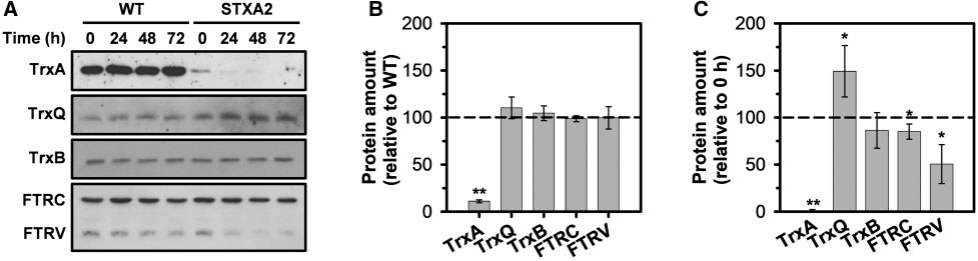
Effect of TrxA levels on the FTR-Trx system proteins in the STXA2 strain. A, Western blot analysis of TrxA, TrxQ, TrxB, FTRC, and FTRV before (0 h) and after inducer removal (24–72 h). Samples equivalents to 4 ×10^7^ cells of WT and STXA2 were taken at the indicated times and total proteins were isolated and resolved on SDS–PAGE, blotted and incubated with specific antibodies indicated in Supplemental Table S2. B, Quantification of the different proteins in the STXA2 strain before inducer removal (0 h) relative to the WT (dashed line). **P <* *0.01 (two-tailed Student’s *t* test between WT and STXA2 strains). C, Quantification of the indicated proteins in the STXA2 strain after 48 h without inducer relative to the signal with inducer (0 h; dashed line). Quantifications were made using ImageJ software. Data are means ± sd from three biological replicates in all cases. *P <* *0.05 and **P <* *0.01 (two-tailed Student’s *t* test between 0 and 48 h).

To evaluate the influence of TrxA decrease on redox regulation in *Synechocystis*, we decided to analyze the levels of the components of FTR-Trx system by immunoblotting in WT and STXA2 strains before (0 h) and after inducer removal (24–72 h; Figure 2). Levels of the thioredoxins TrxQ and TrxB and the FTR, catalytic subunit (FTRC) and variable subunit (FTRV), did not change significantly in STXA2 relative to the WT in the presence of inducer (Figure 2, A and B). After 48 h of inducer removal, the levels of TrxQ increased in STXA2 and reached 150%, while the levels of FTRV decreased and reached 50% relative to the initial time point (Figure 2, A–C).

### Depletion of TrxA strongly affects the photosynthetic activity

**Figure 3:**
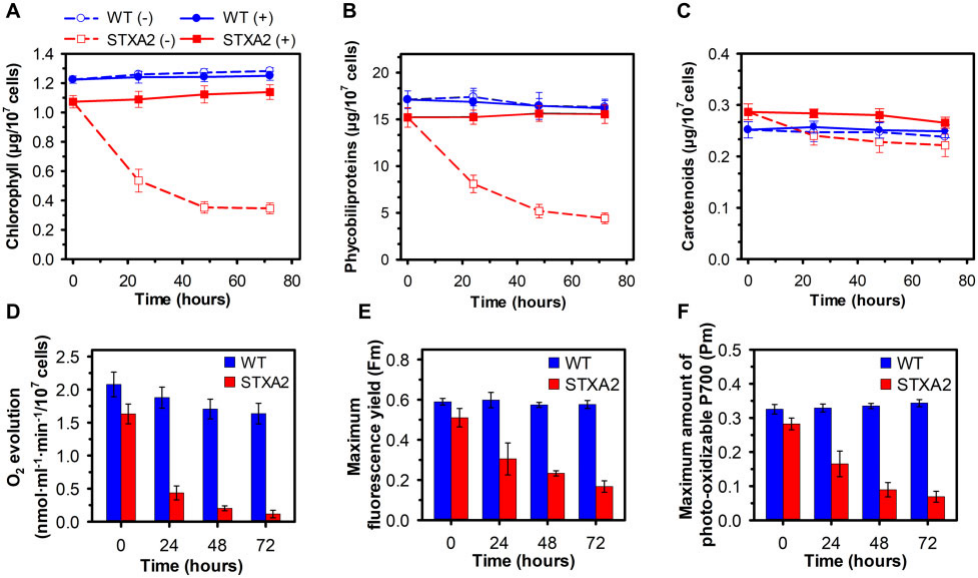
Effects of TrxA levels on photosynthetic pigments, oxygen production and PSII and PSI maximum yield in the STXA2 strain. A, Chl, (B) PBS, and (C) carotenoids contents in the WT and STXA2 strains in the presence (+) or absence of inducer (−; 24–72 h). D, Oxygen evolution rates under growth light (50 µmol photons·m^−2^·s^−1^) measured on a Clark-type electrode. E, Maximum fluorescence yield (*F*m) determined in the presence of DCMU (20 µM) under growth light (50 µmol photons·m^−2^·s^−1^). F, Maximum amount of photo-oxidizable P700 (*P*m) determined as the change in absorbance at 830 nm relative to absorbance at 875 nm after applying a saturation pulse (5,000 µmol photons·m^−2^·s^−1^, 200 ms) in cells pre-illuminated with far-red light (720 nm, 75 W·m^−2^). Data are means ± sd from three biological replicates in all cases.

The observation that the STXA2 strain has a severely altered phenotype in the absence of inducer led us to investigate the effect of TrxA on photosynthetic activity. We initially determined the cellular pigment content in STXA2. In the presence of inducer, both chlorophyll (Chl) and phycobiliproteins (PBS) contents were reduced by 20% in STXA2, while the carotenoid content increased by 12%, as compared to the WT (Figure 3, A–C). Chl and PBS gradually decreased in STXA2 after inducer removal (Figure 3, A and B), and both Chl and PBS contents were reduced by 60%–70% relative to the initial values at 48 h. Interestingly, carotenoids content was less affected than Chl content and an increase in carotenoids/Chl ratio was observed in STXA2 at 48 h (Supplemental Figure S2). Accordingly, the photosynthetic oxygen-evolving rate under growth light intensity was reduced by 88% in STXA2 after 48 h of inducer removal (Figure 3D). In addition, the photosynthetic oxygen-evolving rates were measured at different light intensities and the maximum rate of oxygen evolution in STXA2 was also much lower after 48 h of inducer removal (Supplemental Figure S3A). In contrast, the oxygen consumption rate in the dark due to respiratory processes was higher in STXA2 than the WT in all conditions (Supplemental Figure S3B). We also measured the maximum fluorescence yield in the light when electron transfer is blocked by the inhibitor DCMU (*F*m). In cyanobacteria, this fluorescence estimated by PAM fluorometer is emitted from Chl and PBS (Campbell et al., 1998). Additionally, DCMU is needed to obtain the maximum size of the PSII complex because both respiratory and photosynthesis occur in the thylakoid membranes. The *F*m decreased over time in STXA2 strain after inducer removal (Figure 3E). Moreover, the maximum amount of photo-oxidizable P700 (*P*m), which indicates the fractions of functional PSI in the cells, also decreased in STXA2 (Figure 3F). Because about 80% of Chl in *Synechocystis* is associated with PSI (Shen et al., 1993), we also measured the *P*m on the per Chl basis. In this case, the changes in *P*m only were visible after 48 h of inducer removal, when TrxA levels were < 2%, indicating that the low amount of photo-oxidizable P700 is not solely due to the reduced number of PSI (Supplemental Figure S3, C and D).

**Figure 4:**
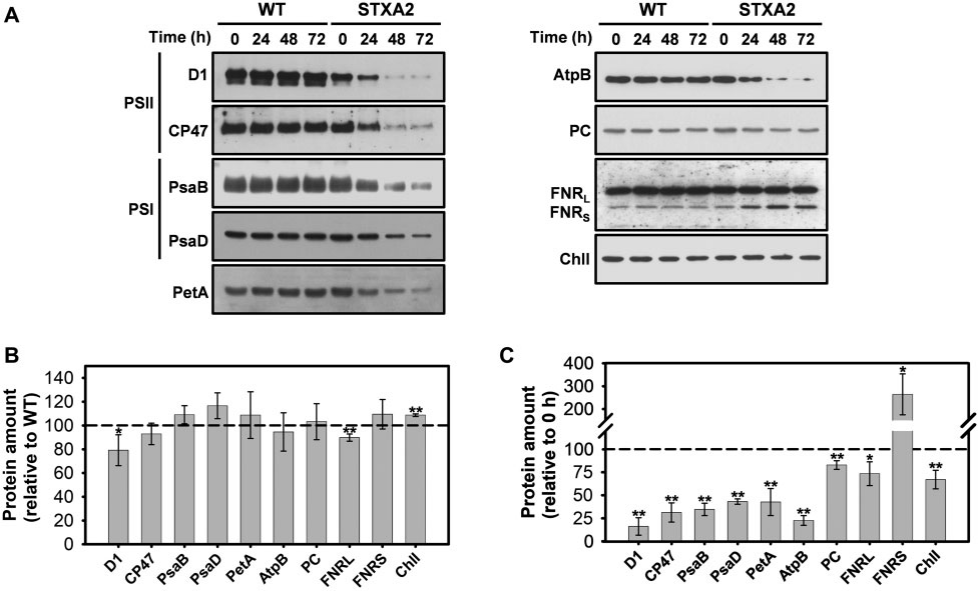
Effects of TrxA levels on photosynthetic-related proteins in the STXA2 strain. A, Western blot analysis of representative components of photosynthetic electron transport, ATP synthase, and chlorophyll synthesis in WT and STXA2 strains. Samples equivalents to 4 ×10^7^ cells were taken before (0 h) and after inducer removal (24–72 h). Total proteins were isolated and resolved on SDS–PAGE, blotted and incubated with specific antibodies. B, Quantification of the indicated proteins in the STXA2 strain before inducer removal (0 h) relative to the WT (dashed line). *P <* *0.05 and **P <* *0.01 (two-tailed Student’s *t* test between WT and STXA2 strains). C, Quantification of the indicated proteins in the STXA2 strain after 48 h without inducer relative to the signal with inducer (0 h; dashed line). Quantifications were made using ImageJ software. Data are means ± sd from three biological replicates in all cases. *P <* *0.05 and **P <* *0.01 (two-tailed Student’s *t* test between 0 and 48 h).

We analyzed whether the decrease in photosynthetic electron transport was accompanied by changes in the abundance of photosynthesis-related proteins. We had previously observed that total protein of WT and STXA2 strains were differentially affected after inducer removal. Thus, the WT strain maintained almost the same protein amount along time whereas STXA2 decreases up to two-fold in 48 h (Supplemental Figure S4). Western blot analyses were performed before (0 h) and after inducer removal (24–72 h). In STXA2, protein levels showed only slight changes relative to the WT in the presence of inducer (Figure 4, A and B), while the inducer removal led to strong changes in protein levels. In addition, we compared the protein levels after 48 h of inducer removal with the initial time point. The content of D1 and CP47 subunits of the PSII complex were reduced to 16% and 31% of the initial value, respectively (Figure 4, A–C). The levels of PsaB and PsaD subunits of the PSI complex decreased to approximately 35% and 43% of the initial value, respectively (Figure 4, A–C). The amount of PetA (Cytochrome f of the Cytochrome b6f complex), AtpB (beta subunit of ATP synthase), and Plastocyanin (PC), which is a soluble electron carrier between the Cytochrome b6f and PSI complexes, were reduced to approximately 43%, 23%, and 85% of their initial values, respectively (Figure 4, A–C). The large form of ferredoxin-NADP+ oxidoreductase (FNR) was moderately decreased to 73%, while the small form of FNR, which has been proposed to participate in cyclic electron transport (CET) by oxidizing NADPH (Thomas et al., 2006; Korn et al., 2009), was increased by 164% of the initial values (Figure 4, A–C). Interestingly, the amount of the catalytic I subunit of magnesium chelatase (ChlI) also was reduced to 70% of the initial value (Figure 4, A–C). Overall, the protein accumulation and pigment content were not enough to sustain photosynthesis electron transport under standard conditions in STXA2 when TrxA levels were below 10% that of the WT.

### Changes in CBB cycle activation and redox state of FBP/SBPase

**Figure 5:**
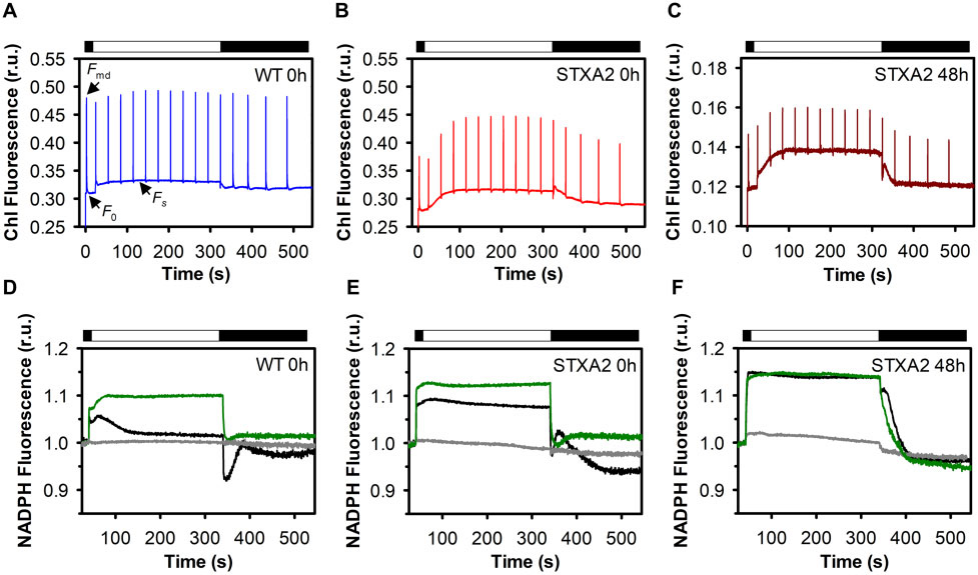
Responses of Chl and NADPH kinetics to changes in TrxA levels. Fluorescence induction curves in (A) WT and (B) STXA2 in the presence of inducer, and (C) STXA2 after 48 h of inducer removal. Cells were dark adapted for 15 min and then exposed to actinic light of 50-µmol photons m^−2^·s^−1^ for 5 min. Saturating pulses of 10,000 µmol photons m^−2^·s ^−1^ (200 ms) were applied every 30 s. The cultures were adjusted to 4 ×10^7^ cells before the measurements. NADPH fluorescence in (D) the WT and (E) STXA2 in the presence of inducer, and (F) STXA2 after 48 h of inducer removal. Cells were dark adapted for 15 min in the absence of inhibitor (black traces) and in the presence of 20-μM DCMU (gray traces) or 10-mM GA (green traces), then exposed to red actinic light of 50-µmol photons m^−2^·s ^−1^ for 5 min. Data are means ± sd from three biological replicates in all cases. r.u., relative units.

To understand the underlying mechanism for the specific role of TrxA in adjusting photosynthetic performance, we analyzed time-resolved changes in Chl and NADPH fluorescence of the WT and STXA2 in the presence of inducer, and after 48 h of inducer removal (Figure 5). The measurements were performed during 5 min illumination with actinic light (50 μmol photons·m^−2^·s^−1^) of dark-adapted cells. Since the respiratory and photosynthetic electron transport chains in cyanobacteria are in the thylakoid membranes, the PQ pool is reduced in the dark (Aoki and Katoh, 1982; Mullineaux and Allen, 1986). In STXA2 with the inducer, Chl fluorescence analysis showed substantially lower dark maximal fluorescence (*F*md) than in the WT (Figure 5, A and B). In addition, the dark *F*vd/*F*o, which indicates the redox state of the PQ pool (Calzadilla et al., 2019), was also smaller in STXA2 (Table 1). These data suggest that the PQ pool was more strongly reduced in dark-adapted STXA2 than in the WT, probably because its reduction via NAD(P)H dehydrogenase-like (NDH-1) is faster than its oxidation by respiratory terminal oxidases. When TrxA levels are <2% in STXA2 relative to the WT, the total fluorescence signal was much lower and higher differences were observed in the dark *F*vd/*F*o (Figure 5C,Table 1).

**Table 1.** Changes in the dark maximal fluorescence (*F*md), the variable fluorescence in darkness/basal fluorescence in darkness (*F*vd/*F*o), ratio and the effective PSII yield (YII) in WT and STXA2 strains. The cultures were adjusted to 4 ×·107 cells before the measurements. Y(II) values were measured from cultures grown at growth light conditions (50 µmol photons m^−2^·s ^−1^) in the WT and STXA2 in the presence of inducer (0 h), and STXA2 after inducer removal (48 h). Data are means ± sd from three biological replicates in all cases.

Strain	*F*md	*F*vd/*F*o	Y(II)
WT 0 h	0.471 ± 007	0.535 ± 0.027	0.319 ± 0.009
STXA2 0 h	0.367 ± 011	0.333 ± 0.008	0.283 ± 0.005
STXA2 48 h	0.147 ± 012	0.221 ± 0.021	0.138 ± 0.022

Upon illumination with actinic light, a rapid increase in the basal fluorescence (*F*s) levels was observed in the WT (Figure 5A). This increase depends on state transitions and CBB cycle activation (Holland et al., 2015). In STXA2, a progressive increase was observed, which was more pronounced after inducer removal (Figure 5, B and C). The *F*s remained low compared to light maximal fluorescence (*F*m′) in the WT, which corresponds to a largely oxidized PQ pool under growth light intensity (Figure 5A). This difference was slightly lower in STXA2 in the presence of inducer than in the WT, and was drastically lower after inducer removal (Figure 5, B and C). In addition, the effective quantum yield of PSII (YII) dropped in STXA2 after inducer removal (Table 1). Concomitantly, NADPH accumulated upon dark to light transition in all cases (Figure 5, D–F). In the WT, the levels of NADPH begin to decline after approximately 30 s (Figure 5D), which has been attributed to the activation of the CBB cycle (Holland et al., 2015). This is supported by the observation that the inhibition of the CBB cycle with glycolaldehyde (GA) abolished this decrease (Figure 5D). In STXA2 with the inducer, very little oxidation of the NADPH pool occurred during illumination and fluorescence reached values greater than that of the WT (Figure 5, D and E). The decrease of TrxA levels in STXA2 after inducer removal led to an increase in the steady-state level of NADPH fluorescence, similar to the values obtained in the presence of GA (Figure 5F).

The transient increase of Chl fluorescence after illumination is due to the reduction of the PQ pool by NADPH or other reducing equivalents accumulated in the light (Mi et al., 1995; Xu et al., 2016). As described previously (Holland et al., 2015; Xu et al., 2016), the WT showed a fast phase (P1) followed by one slow phase (P2) (Supplemental Figure S5A). In the presence of inducer, both phases were higher in STXA2 than in the WT (Supplemental Figure S5A). After 48 h of inducer removal, P1 remained while P2 disappeared in STXA2 (Supplemental Figure S5A). Because the fast and slow phases are related to the NDH-1 and electron donation from NADPH and substrates produced in the CBB cycle, respectively (Holland et al., 2015; Xu et al., 2016), we decided to assess the effect of GA on the transient increase of fluorescence (Supplemental Figure S5B). The fast phase was stimulated while the slow phase was suppressed in WT and STXA2 strains with the inducer, indicating an increase in the electron donation directly to the PQ pool by NDH-1 complexes when the CBB cycle is inhibited (Supplemental Figure S5B). Additionally, the slow phase coincided with a transient re-reduction of the NADP+ after turning off the actinic light (Figure 5, D–F). In the WT, this re-reduction reached maximum NADPH fluorescence 45 s after cessation of illumination, while STXA2 with the inducer reached it at 20 s (Figure 5, D and E). In STXA2 after inducer removal, the re-reduction of the NADP+ was diminished and was shifted to earlier times (Figure 5F).

All these data reflect an increase in the CET around PSI possibly due to an imbalance between photosynthesis and CBB cycle. The CET around PSI acts as an antioxidant mechanism by reducing ROS production (Zhang et al., 2020). As the slower electron transfer from PSII may be related to a more reduced electron chain, we monitored the redox state of P700 during light–dark transitions by the application of actinic light in the presence of inhibitors. DCMU and KCN were used to distinguish between linear electron transport (LET) from PSII, CET around PSI, and electron drain to respiration via oxygen-utilizing electron sinks such as cytochrome c oxidase (Cox) and cytochrome bd quinol oxidase (Cyd), which also contribute to the alleviation of excess electrons in the light (Ermakova et al., 2016; Supplemental Figure S6A). Based on P700 re-reduction (Supplemental Figure S6B), electron transport rates were determined for the different routes (Supplemental Figure S6C). In STXA2 with the inducer, the contribution of CET and Cyd/Cox were increased by 80% and 130%, respectively, whereas LET was decreased by 35% in comparison to the WT (Supplemental Figure S6C). After 48 h of inducer removal, electron flow changed considerably in the STXA2 strain. CET and Cyd/Cox were increased over 140% and 170%, respectively, whereas LET decreased over 66% relative to the initial values (Supplemental Figure S6C). Overall, our results clearly indicate that a deficiency in the amount of TrxA affects the functioning of the CCB cycle, leading to changes in electron flux and suggesting that the primary cause for the strong impairment of photosynthesis is a limitation of the CBB cycle.

**Figure 6:**
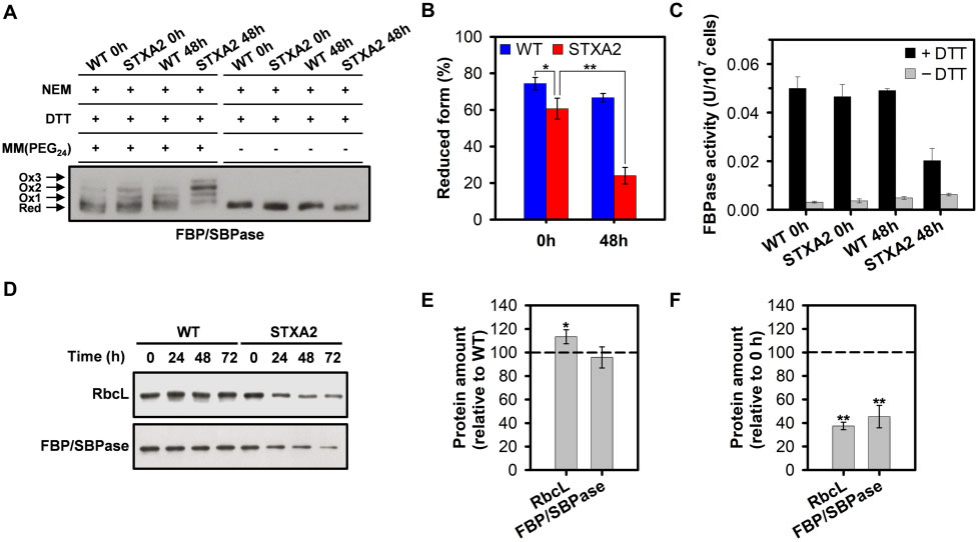
Changes in CBB protein levels and in vivo redox state of FBP/SBPase in response to different TrxA levels. A, In vivo redox state of FBP/SBPase in WT and STXA2 strains. Proteins were extracted from cells before (0 h) and after 48 h of inducer removal (48 h) and labeled with the alkylating agent MM(PEG_24_) as described in the “Materials and methods”. Red, reduced form; Ox, oxidized form. B, Reduction level of FBP/SBPase is defined as the ratio of the reduced form and the sum of reduced and oxidized forms. C, In situ FBPase activity from WT and STXA2 cells growing under standard conditions before (0 h) and after 48 h of inducer removal (48 h). DTT 10 mM was added to reduce the enzyme and determine the maximal activity. D, Western blot analysis of the RbcL and FBP/SBPase before (0 h) and after inducer removal (24–72 h). Samples equivalents to 4 ×10^7^ cells were taken at the indicated times and total proteins were isolated and resolved on SDS–PAGE, blotted and incubated with specific antibodies referenced in Supplemental Table S2. E, Quantification of the indicated proteins in the STXA2 strain before inducer removal (0 h) relative to the WT (dashed line). *P <* *0.05 (two-tailed Student’s *t* test between WT and STXA2 strains). F, Quantification of the indicated proteins in the STXA2 strain after 48 h inducer removal relative to the signal with inducer (0 h; dashed line). Data are means ± sd from three biological replicates in all cases. **P <* *0.01 (two-tailed Student’s *t* test between 0 and 48 h).

As previously mentioned, some CBB enzymes in plants have not an evolutive origin related to cyanobacteria, two of these are the FBPase and SBPase enzymes (Jiang et al., 2012; Gütle et al., 2016). In cyanobacteria, FBP/SBPase activities reside in the same protein and its redox regulation by Trx is not well established in vivo. The FBPase activity in *Synechococcus* sp. 6301 and *Synechocystis* is under the control of oxidizing and reducing conditions (Udvardy et al., 1982; Feng et al., 2014), and is activated in vitro by cyanobacterial *m*-type Trx (Schmidt, 1980; Yee et al., 1981; Crawford et al., 1984; Feng et al., 2014). *Synechocystis* FBP/SBPase has nine cysteine residues of which at least three cysteine residues (C75, C84, and C99) are redox sensitive and that could correspond to those previously identified in structural and redox proteomic studies based on light/dark cycles (Ansong et al., 2014; Guo et al., 2014). In order to determine the in vivo redox state of FBP/SBPase in WT and STXA2 strains before and after 48 h of inducer removal, we used the alkylating agent methyl-PEG24-maleimide (MM(PEG)_24_) as detailed in the “Materials and methods”. Western blot analysis showed up to three shifted bands corresponding to different oxidized forms (Figure 6A). In the presence of inducer, the reduced form of FBP/SBPase was predominant in both strains but lower in the STXA2 mutant (Figure 6, A and B). The reduced form represents 75% of the total protein in the WT, while it was 60% in STXA2. In contrast, the reduced form substantially decreased to 24% of the total protein in STXA2 after inducer removal (Figure 6, A and B). In addition, the oxidized form (Ox2) predominated in STXA2 after 48 h of inducer removal which might be due to a disulfide bridge (Figure 6A). The FBPase activity measured in situ showed higher activity under reducing conditions (dithiothreitol [DTT]) in WT and STXA2 cells (Figure 6C), reflecting the reductive activation of this enzyme. The levels of FBPase activity with DTT were lower and related to levels of FBP/SBPase protein in STXA2 after inducer removal. In cell crude-extracts of both WT and STXA2 strains, DTT activation of the enzyme was confirmed, as the same occurs using TrxA and low amount of DTT (Supplemental Figure S7). Finally, the levels of FBP/SBPase along with RbcL, the large subunit of Rubisco, decreased to 46% and 38% relative to the initial time point, respectively, after 48 h of inducer removal in the STXA2 strain (Figure 6, D–F).

### Interplay among TrxA, ROS, and redox state of 2-Cys Prx

**Figure 7:**
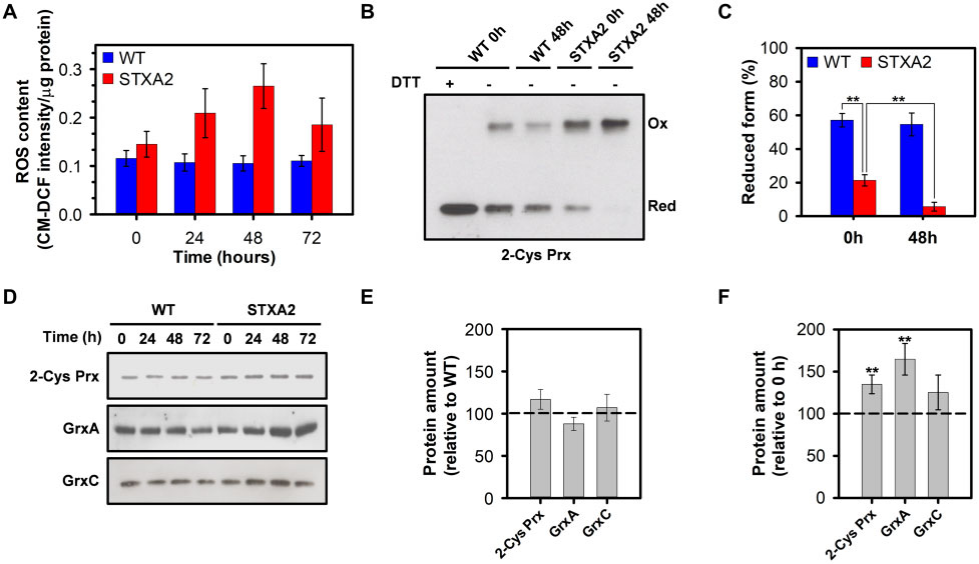
Sensitivity and response to ROS of WT and STXA2 strains. A, Time course of relative ROS amount in the WT and STXA2 before (0 h) and after inducer removal (24–72 h) using the CM-H_2_DCFDA reagent. B, In vivo redox state of 2-Cys Prx in the WT and STXA2 strains. Cells were collected before (0 h) and after 48 h of inducer removal (48 h). The pellets were resuspended in 1× Laemmli buffer and boiled for 10 min, then 20 μL of the boiled cell suspension were loaded on a SDS–PAGE gel under nonreducing conditions, except for a sample of the WT strain that was also treated with DTT 10 mM as a control. 2-Cys Prx was detected by Western blot using specific antibody. C, Reduction level of 2-Cys Prx is defined as the ratio of the reduced form (monomer) and the sum of reduced and oxidized forms (monomer and dimer respectively). D, Western blot analysis of GrxA, GrxC, and 2-Cys Prx before (0 h) and after inducer removal (24–72 h). Samples equivalents to 4 ×10^7^ cells were taken at the indicated times and total proteins were isolated and resolved on SDS–PAGE, blotted and incubated with specific antibodies. E, Quantification of the indicated proteins in the STXA2 strain before inducer removal (0 h) relative to the WT (dashed line). F, Quantification of the indicated proteins in the STXA2 strain after 48 h inducer removal relative to the signal with inducer (0 h; dashed line). Data are means ± sd from three biological replicates in all cases. **P <* *0.01 (two-tailed Student’s *t* test between 0 and 48 h).

Cyanobacteria and other oxygenic photosynthetic organisms produce ROS in the light that potentially affects the photosynthetic machinery. Thus, these organisms maintain an adjusted redox balance and control ROS using multiple systems, including modulation of phycobilisomes and carotenoids relative to Chl, enzymatic ROS scavenging and redox control (Latifi et al., 2009; Pathak et al., 2019). Because the phenotype observed in STXA2 could be a consequence of an increase in ROS generation, we analyzed the ROS content in the WT and STXA2 strains before (0 h) and after inducer removal (24–72 h) using the ROS marker CM-H_2_DCFDA. In the presence of inducer, STXA2 had 26% higher ROS content than the WT (Figure 7A). The ROS content increased in STXA2 until 48 h after inducer removal, where it was about 85% higher than the initial level (Figure 7A). Since the ROS generation under normal conditions occurs via photosynthetic electron transport chain, we tested the response of the WT and STXA2 strains to the addition of 5-µM methyl viologen (MV), a PSI electron acceptor that produces ROS upon oxidation, before and after 48 h of inducer removal. In the presence of inducer, the MV treatment led to an increase in ROS content that was two-fold higher than the control in the WT and 3.5-fold higher in STXA2 (Supplemental Figure S8A). The results were similar after 48 h of inducer removal (Supplemental Figure S8A), possibly due to the decrease in photosynthesis observed at this point in STXA2. Overall, these results suggest that the observed differences are not only because of the limitation in the CBB cycle but also due to possible imbalances in some protective mechanisms. In cyanobacteria, Prxs are particularly important in the response to oxidative stress generated inside the cells or by the exogenous addition of hydrogen peroxide (Dietz, 2011). Because one of these Prxs, 2-Cys Prx, has been extensively studied in *Synechocsytis* and could receive reducing equivalents from TrxA in vitro (Hosoya-Matsuda et al., 2005; Pérez-Pérez et al., 2009b), we analyzed the in vivo redox state of 2-Cys Prx in WT and STXA2 strains before and after 48 h of inducer removal (Figure 7B). In the presence of inducer, the reduced form that is detected as a monomer band, represents 65% of the total protein in the WT, while it was 20% in STXA2 (Figure 7, B and C). In addition, the reduced form was almost undetectable in STXA2 after 48 h of inducer removal (Figure 7, B and C). These results indicate that the redox state of 2-Cys Prx depends directly on TrxA levels, suggesting that TrxA is the main donor for this Prx in vivo. Additionally, we analyzed the levels of 2-Cys Prx and two glutaredoxins, GrxA and GrxC, that are essential for stress adaptation in cyanobacteria (Sánchez-Riego et al., 2013). Only levels of the 2-Cys Prx were slightly increased in STXA2 with the inducer compared to the WT (Figure 7, D and E). In contrast, the amount of 2-Cys Prx increased by 30%, whereas GrxA and GrxC increased by 70% and 20%, respectively, in STXA2 after 48 h of inducer removal (Figure 7, D–F). To test the antioxidant significance of TrxA deficiency in STXA2, we investigated the effect of H_2_O_2_ addition on cell viability. Different concentrations of H_2_O_2_ were added to the cultures under induction or after 48 h of inducer removal (Supplemental Figure S8B). In the presence of inducer, STXA2 showed more sensitivity to the H_2_O_2_ treatment than the WT and was unable to grow at 1.5 mM of H_2_O_2_, while the WT showed only strong differences at 3 mM of H_2_O_2_ (Supplemental Figure S8B). All H_2_O_2_ concentrations tested were lethal in STXA2 after 48 h of inducer removal (Supplemental Figure S8B).

## Discussion

In cyanobacteria, TrxA has been shown to be essential (Muller and Buchanan, 1989; Navarro and Florencio, 1996), and therefore mutants lacking this protein are not available. In addition, TrxA is about 10 and 50 times more abundant than TrxB and TrxQ, respectively, in normal growth conditions (Florencio et al., 2006). In our study, we were able to obtain a strain of *Synechocystis* (STXA2) with a low level of TrxA, ranging from 10% to 2% relative to the WT, depending on the presence or absence of an inducer. The STXA2 mutant grows almost as well as the WT with only 10% of TrxA, but only when this TrxA level decreased a drastic phenotype emerged (Figures 1 and 2). These results reinforce the idea that TrxA is an essential protein in cyanobacteria. In *Anabaena* only the mutant lacking Trx-*m*1 showed growth arrest under diazotrophic conditions, but it is not reported what specific function fails (Mihara et al., 2018). In Arabidopsis the *trx m124-2* triple mutant was affected in growth, although the amount of total Trx-*m* was 23%. These mutants did not accumulate Trx-*m*4, which could explain this phenotype.

STXA2 has stronger phenotype with 2% of TrxA and the analysis of other redox proteins shows that only TrxQ levels increased (Figure 2). As TrxQ appears to have a role against oxidative stress (Pérez-Pérez et al., 2009a), it could be upregulated in response to oxidative stress following TrxA depletion. Despite this, TrxQ levels are more than 50 times less abundant than TrxA levels (Florencio et al., 2006). On the other hand, we observed a decrease in the levels of FTR subunits (Figure 2). This effect could respond to changes in TrxA levels, taking into account that this Trx is probably its more abundant substrate. Although changes in the levels of both FTR subunits could affect the complex, FTRV is apparently not essential for cell viability in *Synechocystis* (Hishiya et al., 2008). The FTRV subunit appears to have a structural role protecting the Fe-S cluster of the FTRC from oxygen (Balsera et al., 2013) and does not participate in the catalytic reaction (Dai et al., 2000, 2007). Furthermore, it is probably less expensive to degrade the FTRV than the FTRC subunit, since the last one has the additional cost of the Fe-S center.

Decrease in TrxA levels by 90% showed no major changes in photosynthetic activity, pigments and photosynthesis-related proteins in STXA2 (Figures 3 and 4). However, Chl and NADPH fluorescence kinetics revealed changes during dark–light transitions and at steady state (Figure 5). STXA2 displayed slightly lower effective PSII yield (Table 1), a more reduced NADPH pool and changes in electron flux (Figures 5;Supplemental Figure S5). Previous studies have reported that the inorganic carbon limitation or inhibition of the CBB cycle in *Synechocystis* increases the NADPH fluorescence during illumination (Holland et al., 2015; Xu et al., 2016). This is due to a hindered CBB cycle resulting in less NADPH oxidizers, which could explain the differences between WT and STXA2 strains. In the STXA2 strain with 10% TrxA, LET from PSII was slightly decreased, while CET around PSI and respiratory pathways, which cooperate to allow maintenance of redox balance between the photosynthesis and CO_2_ fixation, were increased (Supplemental Figure S5). This readjustment in electron transport pathways allows optimizing the relationship between photosynthesis and the CBB cycle (Miller et al., 1988; Badger and Schreiber, 1993), and preventing excess ROS production (Kramer and Evans, 2011). When TrxA levels were decreased <2% of that in the WT, Chl, and NADPH fluorescence kinetics revealed a blockage of downstream electron sinks in the CBB cycle that prevents the oxidation of the PQ pool (Figure 5). Concomitantly, LET decreased and electron flux to the CET and Cyd/Cox increased (Supplemental Figure S5). Analysis of an Arabidopsis mutant lacking Trx-*m*4 showed that this Trx directly downregulates CET activity (Courteille et al., 2013), by forming a complex with PGR5-like photosynthetic phenotype 1 (PGRL1; Okegawa and Motohashi, 2020). Although cyanobacteria have a PGRL1 analog (Dann and Leister, 2019), and we cannot rule out that TrxA is regulating this process, it exhibits weak sequence similarity with the plant PGRL1.

Taking into account the fact that different approaches have identified several key enzymes involved in the CBB cycle in cyanobacteria as modulated by Trx, our attention was focused in FBP/SBPase. We evaluated changes of this CBB enzyme in STXA2 mutant. Our data indicate that FBP/SBPase seems to be mainly reduced under our growth conditions in the WT strain, although various oxidized forms are visible (Figure 6). The oxidized forms increased in the STXA2 strain, where most of the FBP/SBPase remained in the Ox2 redox state after inducer removal (Figure 6). Because this redox state corresponds with a disulfide bridge, it is reasonable to assume that TrxA possibly participates in its reduction. The FBP/SBPase structures from *Synechocystis* and *Thermosynechococcus elongatus* suggested the presence of a disulfide bridge between cysteines 75 and 84 in the inactive state (Feng et al., 2014; Cotton et al., 2015). Despite this, site-directed mutagenesis and activity assays showed another possible disulfide bridge between cysteines 75 and 99 in *Synechocystis* (Feng et al., 2014). In any case, the partial oxidation of FBP/SBPase, which is active in the reduced form and essential for photoautotrophic growth, seems to limit CO_2_ fixation in the STXA2 strain as TrxA levels decrease. These results together with the data obtained for FBPase activity in STXA2 (Figure 6C;Supplemental Figure S7) suggest that TrxA could regulate two key steps of the CBB cycle, FBPase and SBPase activities. This provided evidence for a conserved redox regulation between cyanobacteria and chloroplasts taking in account that the proteins are evolutionarily distinct (Jiang et al., 2012; Gütle et al., 2016). As mentioned, other CBB enzymes such as PRK and CP12 have also been found to be regulated by light/dark cycles. Thus, it cannot be discarded that TrxA also participates in the redox regulation of these proteins in cyanobacteria (Wilson et al., 2019; Yu et al., 2020). Further studies are needed with STXA2 strain to explore the possible redox regulation of other CBB cycle-related proteins by TrxA.

In light, the limitation of the CBB cycle promotes the ROS generation by the photosynthetic electron transport, which causes damage to pigments and proteins (Nishiyama et al., 2006; Latifi et al., 2009). The STXA2 strain had a decreased photosynthetic protein content and accumulated higher ROS levels after inducer removal (Figures 4 and 7). Multiple mechanisms contribute to maintain cellular redox balance and avoid associated light-induced damage (Das and Roychoudhury, 2014; Pathak et al., 2019). Prxs such as 2-Cys Prx are involved in the detoxification response and are known as Trx-dependent peroxidases in cyanobacteria and chloroplast (Dietz, 2011). 2-Cys Prx is efficiently reduced by the three canonical Trxs and interacts in vitro with TrxA and TrxQ (Pérez-Pérez et al., 2009b). In addition to the Trx system, many cyanobacteria possess a NTRC that transfers reducing equivalents to 2-Cys Prx (Banerjee et al., 2015; Sánchez-Riego et al., 2016). Moreover, NTRC reduces 2-Cys Prx more efficiently than Trx-*m*1 in *Anabaena* sp. PCC 7120 (Mihara et al., 2016), indicating two evolutionary divergent antioxidant strategies. Our data show that TrxA levels correspond with the accumulation of the oxidized form of 2-Cys Prx in STXA2 compared to the WT (Figure 7). Furthermore, increased levels of TrxQ, GrxA, and GrxC, which have a role against oxidative stress (Pérez-Pérez et al., 2009a; Sánchez-Riego et al., 2013), could be functionally related to the ROS levels.

In summary, our study reveals that TrxA assumes early regulatory functions along the evolution of oxygenic photosynthetic organisms, at least on CBB cycle control and the response to oxidative stress, where FBP/SBPase and 2-Cys Prx could be considered as representative of both processes that affect the photosynthesis functionality. These functions are actually under control of redox partners in land plants, but with many more players, besides *m*-type thioredoxin.

## Materials and methods

### Culture conditions

*Synechocystis* sp. PCC 6803 cells were grown photoautotrophically in BG11 medium (Rippka R, 1988) supplemented with 1 g·L^−1^ NaHCO_3_ (BG11C) in conical flasks with 1% (v/v) CO_2_ in air, under continuous illumination (4000 K LED lights, 50 µmol photons·m^−2^·s^−1^) at 30°C. For plate cultures, BG11C liquid medium was solidified using 1% (w/v) agar. Nourseothricin and spectinomycin/streptomycin were added to a final concentration of 50 μg·mL^−1^ and 2.5 μg·mL^−1^, respectively when required. For inducer removal experiments, exponentially growing cells in 1-mM NaAsO_2_-containing media were harvested by centrifugation (4300*g* for 5 min), washed twice in the corresponding medium, and resuspended in BG11C medium in the presence or the absence of inducer to 0.8·10^7^ cells·mL^−1^. The number of cells per unit of volume was measured with a BD Influx Cell Sorter flow cytometer (Becton Dickinson) by processing 50 μL of a previously diluted culture to optical density at 750 nm of 0.25 (OD_750 __nm_).

### Generation of STXA2 *Synechocystis* strain

The STXA2 conditional mutant was generated by placing the *slr0623* open reading frame (*trxA* gene) under the control of the P_arsB_ promoter with a nourseothricin resistance cassette (*Nat^R^*). For this purpose, a 324-bp fragment containing the *trxA* gene was amplified by PCR using the oligonucleotides TrxA-NdeI and TrxA-NotI (Supplemental Table S1). This fragment was cloned into NdeI-NotI-digested pglnN_ParsB_natR plasmid containing the nonessential *glnN* gene and a *Nat^R^* cassette. The resulting plasmid was used for transformation of the *Synechocystis* WT strain. Subsequently, two DNA fragments of the *trxA* genomic region were amplified by PCR. A 564-bp fragment was obtained using the oligonucleotides sll0586-F1 and TrxA-R1 (Supplemental Table S1), and a 780-bp fragment was obtained using the oligonucleotides sll0585-F1 and sll0585-R1 (Supplemental Table S1). Both fragments were ligated to generate a *trxA* locus containing a BamHI site. The resulting fragment was cloned into pGEMT plasmid. An Sm^r^ Sp^r^ C.S3 cassette (Prentki and Krisch, 1984) from pRL463 was cloned in BamHI site generating the pGQ5.4+ plasmid. The resulting targeting plasmid containing the mutant variant of *trx*A gene was used to transform the parental mutant strain. All DNA constructs were confirmed by DNA sequencing.

### RNA isolation and Northern blot analysis

Total RNA was isolated and extracted as previously described (García-Domínguez and Florencio, 1997). RNA integrity was confirmed by visualization of intact rRNA under UV light. Northern blots were performed as previously described (Giner-Lamia et al., 2014). Probe for northern blot hybridization was obtained by PCR with oligonucleotide pairs (Supplemental Table S1). The filters were reprobed with the constitutively expressed *rnpB* as a control (Vioque, 1992). Hybridization signals were detected with a Cyclone Plus Storage Phosphor System (Perkin-Elmer).

### Cell extract preparation and alkylation assay

For analysis of the abundance of proteins of interest in *Synechocystis* cells grown under different conditions, crude extracts were prepared using glass beads as previously described (Reyes et al., 1995). Protein concentration in cell-free extracts was determined by the method of Lowry, using Bovine Serum Albumin as a standard (Lowry et al., 1951).

Alkylation assays were performed using N-ethylmaleimide (NEM) and MM(PEG)_24_ (Thermo Scientific). NEM was added directly to cells grown before and after inducer removal to a final concentration of 10 mM. After 1 h of incubation, samples were centrifuged (4,300*g* for 20 min), resuspended in buffer A (50-mM Tris–HCl, pH 8.0, 50-mM NaCl, and 100-mM NEM) and were lysed using glass beads. After centrifugation (15,000*g* for 20 min at 4°C), the supernatants were transferred to a tube and incubated with 10% (w/v) trichloroacetic acid (TCA) for 1 h on ice. Samples were centrifuged (15,000*g* for 20 min), and precipitates were then washed twice with ice-cold acetone and resuspended in buffer B (50-mM Tris–HCl, pH 8.0, 50-mM NaCl, and 2% (w/v) Sodium Dodecyl Sulfate ( SDS) containing 100-mM DTT. After 1 h of incubation at 4°C, samples were treated with TCA and ice-cold acetone again, and precipitates were resuspended in buffer C (50-mM Tris–HCl, pH 8.0, 50-mM NaCl, 2% (w/v) SDS, 7.5% (v/v) glycerol and 0.01% bromophenol blue) with or without 10-mM MM(PEG)_24_.

### Western blot analysis

For western blotting, proteins were resolved by sodium dodecyl–sulfate polyacrylamide gel electrophoresis (SDS–PAGE) according to the method of Laemmli (Laemmli, 1970), transferred to nitrocellulose membranes and immunoblotted with the required antibody in each case using a standard protocol (Supplemental Table S2). ECL Prime Western Blotting Detection Reagent (GE Healthcare) was used to detect the different antigens with antirabbit secondary antibodies (1:50,000; Sigma-Aldrich).

### Pigment analysis

Chl was determined spectrophotometrically at 665 nm in a methanol solution (Mackinney, 1941). For the determination of PBS and carotenoids content, samples were sonicated three times for 60 s each at 70% amplitude on an S-450D Branson Digital Sonifier. Lysates were clarified by centrifugation (3,000*g* for 15 min). PBS and carotenoids contents were determined using the specific absorption coefficient as previously described (Davies, 1976; Siegelman and Kycia, 1978).

### ROS measurements

ROS levels from *Synechocystis* cultures were determined as previously described (Giner-Lamia et al., 2014). Total protein extracts under different conditions were used for ROS determination. A total of 25-μM 5-(and-6)-chloromethyl-2′,7′-dichlorodihydrofluorescein diacetate (CM-H_2_DCFDA) was added to the samples. Then, samples were incubated for 30 min at 30°C and fluorescence was measured with a Varioskan LUX microplate reader (Thermo Scientific) using 485 nm excitation and detecting emission at 525 nm.

### Oxygen evolution

Oxygen evolution was determined on cell cultures using a Clark-type oxygen electrode (Hansatech) maintained at 30°C. Cells were harvested and adjusted to 2 ×·10^7^ cells·mL^−1^ in BG11C medium. The photosynthetic and respiratory rates were recorded for 10 min in the light (50 μmol photons·m^−2^·s^−1^) and the dark, respectively.

### Measurement of Chl and NADPH fluorescence

Both Chl and NADPH fluorescence from intact cells were recorded with a Dual-PAM-100 (Walz) at room temperature. Before measurements, cell suspensions at a cellular concentration of 4 ×·10^7^ cells·mL^−1^ were dark-adapted for 10 min. A 200-ms saturation pulse (5,000 μmol photons·m^−2^·s^−1^) was supplied for the determination of *F*md (maximum fluorescence in darkness), *F*m′ (maximum fluorescence under light) and *F*m (maximum fluorescence in the presence of 20-μM DCMU). Other fluorescence parameters used in the analysis are *F*o (basal fluorescence in darkness) and *F*s (basal fluorescence under actinic illumination). The *F*vd (variable fluorescence in darkness) and Y(II) (effective quantum yield of PSII) were calculated as *F*md − *F*o and (*F*m′−*F*s)/*F*m′, respectively.

The NADPH fluorescence was excited at 365 nm and detected between 420 and 580 nm using the NADPH/9-AA module (Schreiber and Klughammer, 2009).

### P700 measurements

P700 absorption changes were monitored using Dual-PAM-100 (Walz) in the same conditions as described for Chl and NADPH fluorescence measurements. The maximum amount photo-oxidizable P700 (*P*m) was achieved by the application of a saturation pulse under far-red light (720 nm, 75 W·m^−2^). To estimate linear, cyclic and respiratory electron transport rates, cells were exposed to a 30 s growth light intensity and the half-life of P700^+^ reduction was determined in the absence or the presence of 20-μM DCMU and 1-mM KCN using single exponential functions for fitting. Data were interpreted as previously described (Yu et al., 1993; Bernát et al., 2011). In the presence of DCMU, electron transport from PSII is blocked and, therefore, P700^+^ reflects the CET. The difference between the absence and presence of DCMU represents the LET flow. KCN addition on DCMU-treated cells allowed for discrimination between CET and Cyd/Cox-directed electron transport.

### Fructose-1,6-bisphosphatase assay

FBPase activity was determined in situ in permeabilized cells with mixed alkyltrimethylammonium bromide and in cell-free extracts. The standard reaction mixture was containing 150-mM Tris–HCl, pH 8, 15-mM MgSO_4_, and the indicated amounts of DTT and TrxA (10 µM). Samples were incubated at room temperature for 5 min before fructose 1,6-biphosphate was added (7 mM). Then the mixture was incubated in a water bath at 30°C for 15 min. Finally, the reaction was stopped with the Fiske–Subbarow reagent, and the amount of Pi was determined from the absorbance at 660 nm (Wolosiuk et al., 1980). One unit of FBPase activity corresponds to the amount of enzyme that catalyzes the liberation of 1 µmol min^−1^ of Pi.

## Supplemental data

The following materials are available in the online version of this article.

**Supplemental Figure S1.** Construction of STXA2 mutant.

**Supplemental Figure S2.** Analysis of pigment content in WT and STXA2 strains.

**Supplemental Figure S3.** Analysis of photosynthetic and respiratory activities in WT and STXA2 strains.

**Supplemental Figure S4.** Total protein content in WT and STXA2 strains before (0 h) and after inducer removal (24–72 h).

**Supplemental Figure S5.** Change in the Chl fluorescence level in WT and STXA2 strains.

**Supplemental Figure S6.** P700+ re-reduction kinetics and rates in WT and STXA2 strains.

**Supplemental Figure S7.** Reduction of FBP/SBPase by TrxA in WT and STXA2 strains.

**Supplemental Figure S8.** Response to MV and hydrogen peroxide (H_2_O_2_) in WT and STXA2 strains.

**Supplemental Table S1.** Oligonucleotides used in this work.

**Supplemental Table S2.** Antibodies used in this work.

## Supplementary Material

kiab321_Supplementary_Data

## Notes

M.J.M.-P. performed the experiments and analyzed the data. A.M.S.-R. constructed the STXA2 strain. M.J.M.-P., M.J.H., and F.J.F. conceived and designed the experiments. M.J.H. and F.J.F. supervised the work. M.J.M.-P. wrote the article and M.J.H. and F.J.F. contributed and supervised the final article version. F.J.F. agrees to serve as the author responsible for contact and ensures communication.

The author responsible for distribution of materials integral to the findings presented in this article in accordance with the policy described in the Instructions for Authors (https://academic.oup.com/plphys/pages/general-instructions) is: Francisco J. Florencio(floren@us.es).
