# Supplementary material for: Depletion of m-type thioredoxin impairs photosynthesis, carbon fixation, and oxidative stress in cyanobacteria
Source: Plant Physiol. 2021 Jul 15;187(3):1325–40. doi: 10.1093/plphys/kiab321 (PMC8566235; doi:10.1093/plphys/kiab321)
Supplement: kiab321_Supplementary_Data [file kiab321_supplementary_data.pdf]

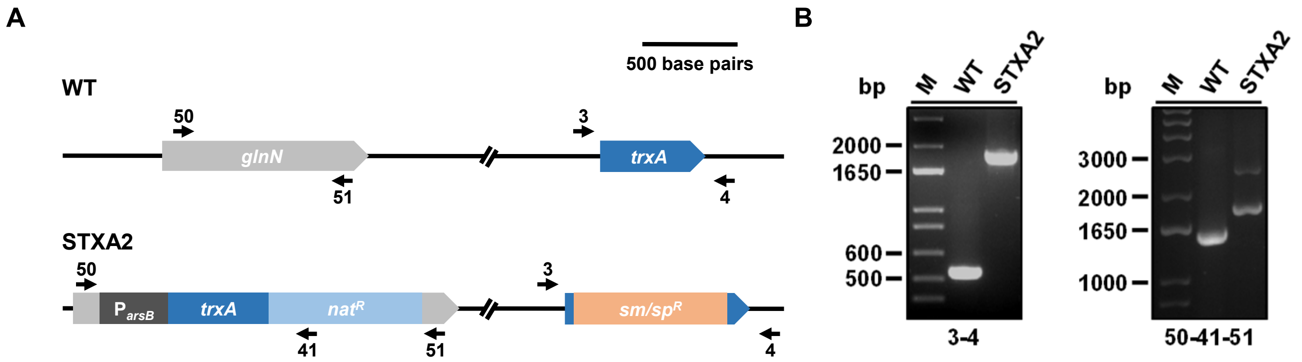

Supplemental Figure S1. Construction of STXA2 mutant. (A) Schematic representation of the *trxA* and *glnN* loci in WT and STXA2 strains. Black arrows represents oligonucleotides used to assess chromosome segregation of P*arsB::trxA::nat* insertion in *glnN* and insertion of Sp/Sm cassette in *trxA* gene. (B) PCR analysis of genomic DNA isolated from WT and STXA2 strains using oligonucleotide pairs as shown in (A).

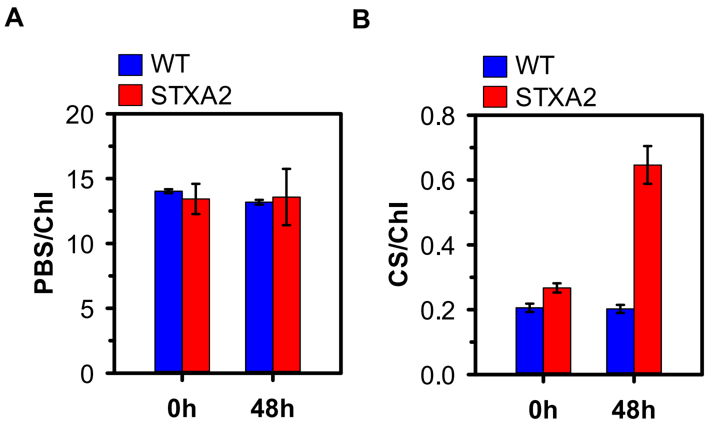

Supplemental Figure S2. Analysis of pigment content in WT and STXA2 strains. Ratios of (A) Phycobiliproteins (PBS)/Chlorophyll (Chl) and (B) Carotenoids (CS)/Chlorophyll (Chl) measured before (0h) and after 48 hours of inducer removal (48h). Data are means ± SD from three biological replicates in all cases.

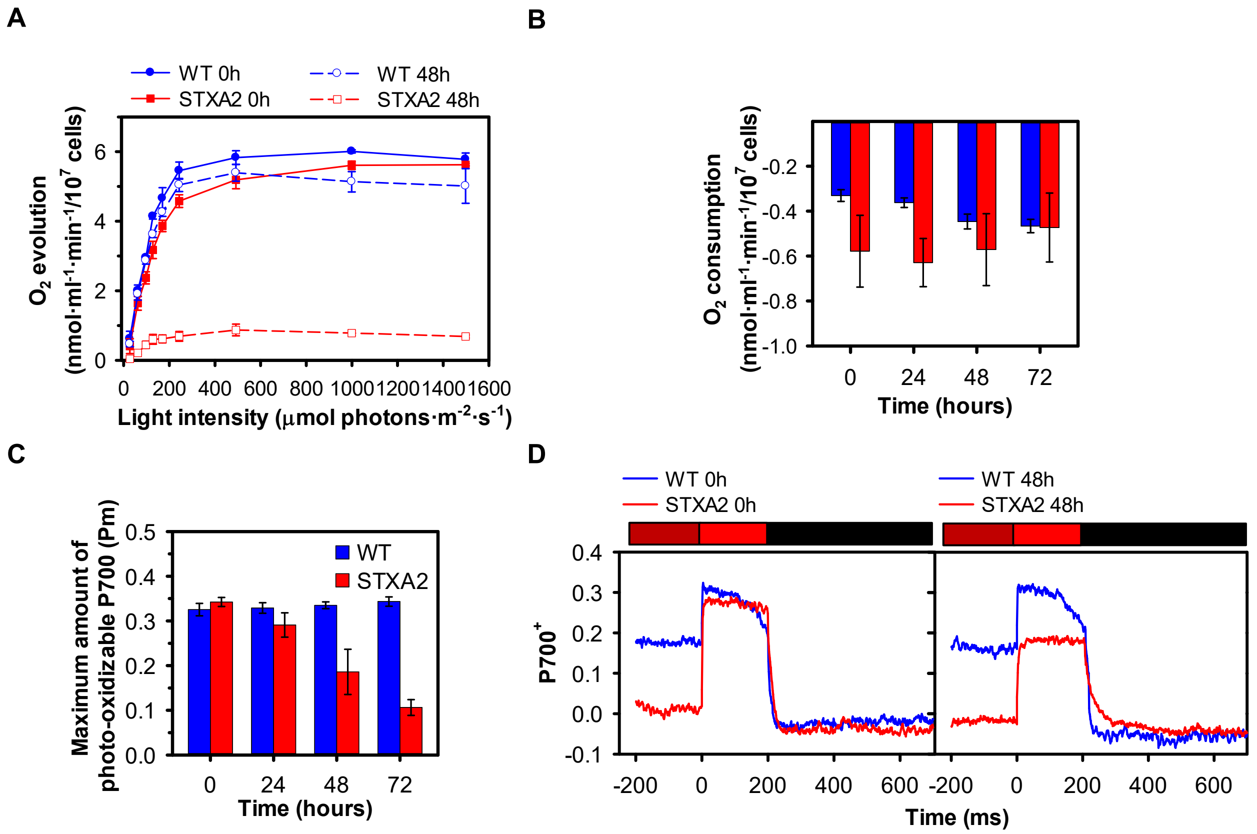

Supplemental Figure S3. Analysis of photosynthetic and respiratory activities in WT and STXA2 strains. (A) Light saturation curve measured before (0h) and after 48h of inducer removal (48h). (B) Time course of oxygen consumption rates before (0h) and after inducer removal (24-72h). WT and STXA2 cells were incubated 10 minutes in the dark and measured on a Clark-type electrode. (C) Time course of maximum amount of photo-oxidizable P700 (Pm) normalized per chlorophyll basis. Pm was determined as the change in absorbance at 830 nm relative to absorbance at 875 nm after applying a saturation pulse (5.000 µmol photons·m^{-2}·s^{-1}, 200 ms) in cells pre-illuminated with far-red light (720 nm, 75 W/m^{-2}). (D) Original traces of $P700^+$ fast kinetics used to obtain the Pm levels in (C). Data are means ± SD from three biological replicates in all cases.

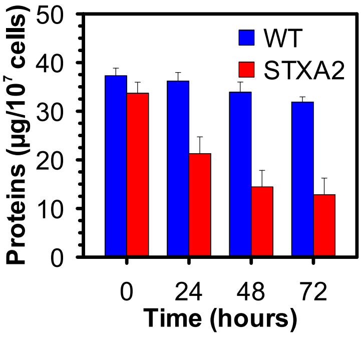

Supplemental Figure S4. Total protein content in WT and STXA2 strains before (0h) and after inducer removal (24-72h). Data are means ± SD from three biological replicates in all cases.

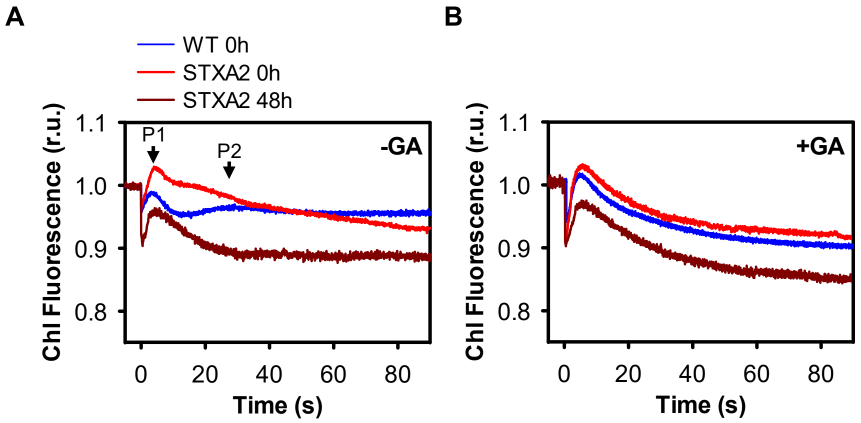

Supplemental Figure S5. Change in the Chl fluorescence level in WT and STXA2 strains. Fluorescence rise measured (A) in the absence or (B) in the presence of glycolaldehyde (GA) from WT and STXA2 strains in presence of inducer and after 48h of inducer removal. The cultures were adjusted to $4 \cdot 10^7$ cells before the measurements. Cells were dark adapted for 15 min and then exposed to red actinic light (AL) of 50 µmol photons $m^{-2} \cdot s^{-1}$ for 5 min. AL was turned off and the subsequent change in the Chl fluorescence level ($F_{0)}$ was monitored as an indicator of PQ pool reduction. The F_0 levels were normalized to the same amplitude to facilitate comparison of the kinetics. P1 represents the reduction of PQ by the cyclic electron flow around PSI and P2 represents the oxidation of sugars accumulated in the cytoplasm during the light period. Data are means ± SD from three biological replicates in all cases. r.u., relative units.

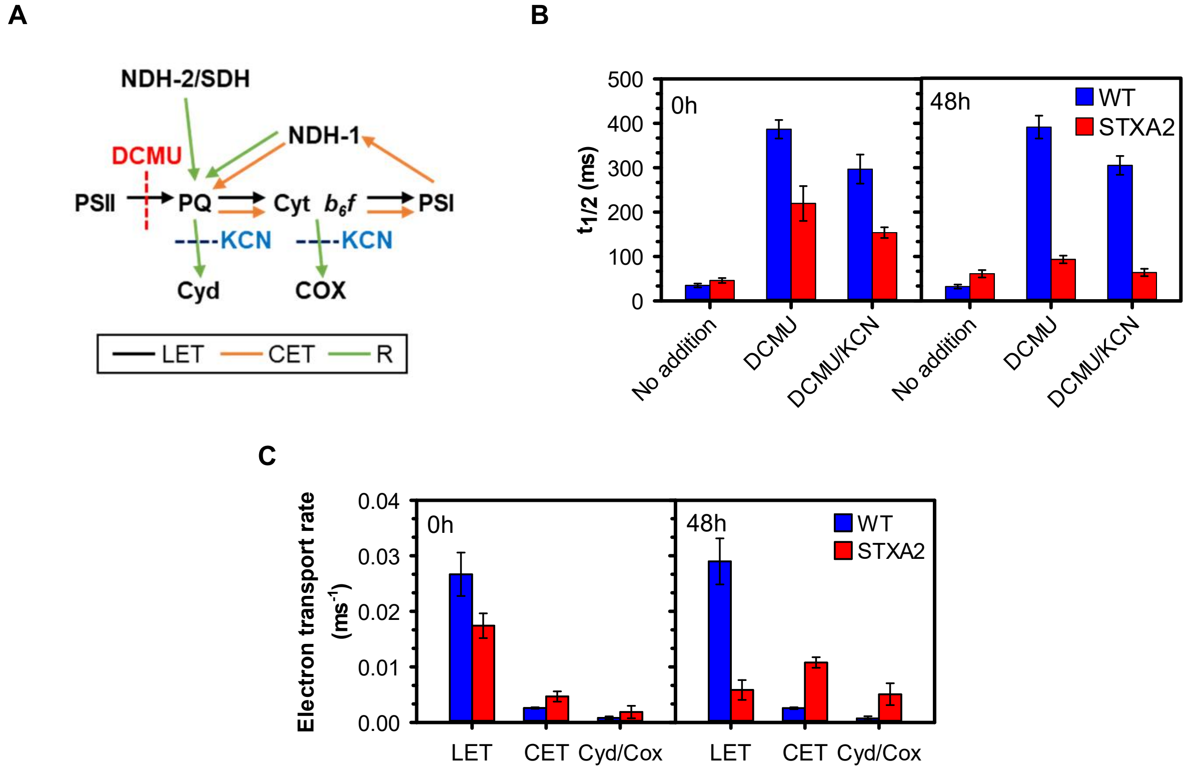

Supplemental Figure S6. P700+ re-reduction kinetics and rates in WT and STXA2 strains. (A) Schematic model for quantification of linear electron transport (LET), cyclic electron transport (CET) and the Cyd/Cox-mediated respiratory flow (Cyd/Cox), which includes the effects of the chemicals 3-(3,4-dichlorophenyl)-1,1-dimethylurea (DCMU) and potassium cyanide (KCN). (B) Half-time of P700+ re-reduction in WT and STXA2 before (0h) and after 48h of inducer removal (48h). Cells were dark adapted for 15 min and then exposed to red actinic light of 50 µmol photons $m^{-2}\cdot s^{-1}$ for 30 s in the absence and presence of inhibitors (DCMU 20 µM or KCN 1 mM). Single exponential functions were used for fitting. (C) Electron transport rates determination for WT and STXA2 in the presence of inductor (0h) or after 48 hours of the inducer removal (48h) based on the kinetic measurements shown in (B). Data are means ± SD from three biological replicates in all cases.

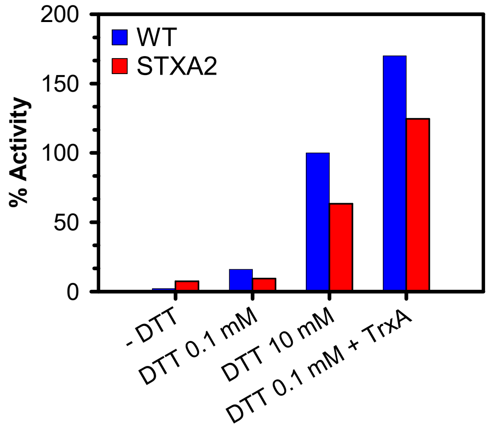

Supplemental Figure S7. Reduction of FBP/SBPase by TrxA in WT and STXA2 strains. FBP/SBPase activity in crude extracts from WT and STXA2 cells growing under standard conditions after 48h of inducer removal. Measurement were performed in absence of DTT, and presence of DTT 0.1 mM, DTT 10 mM and DTT 0.1 mM and TrxA (10 μM). 100% was referred to activity of WT with 10 mM DTT (1.73 U/mg). Data are means from three biological replicates in all cases.

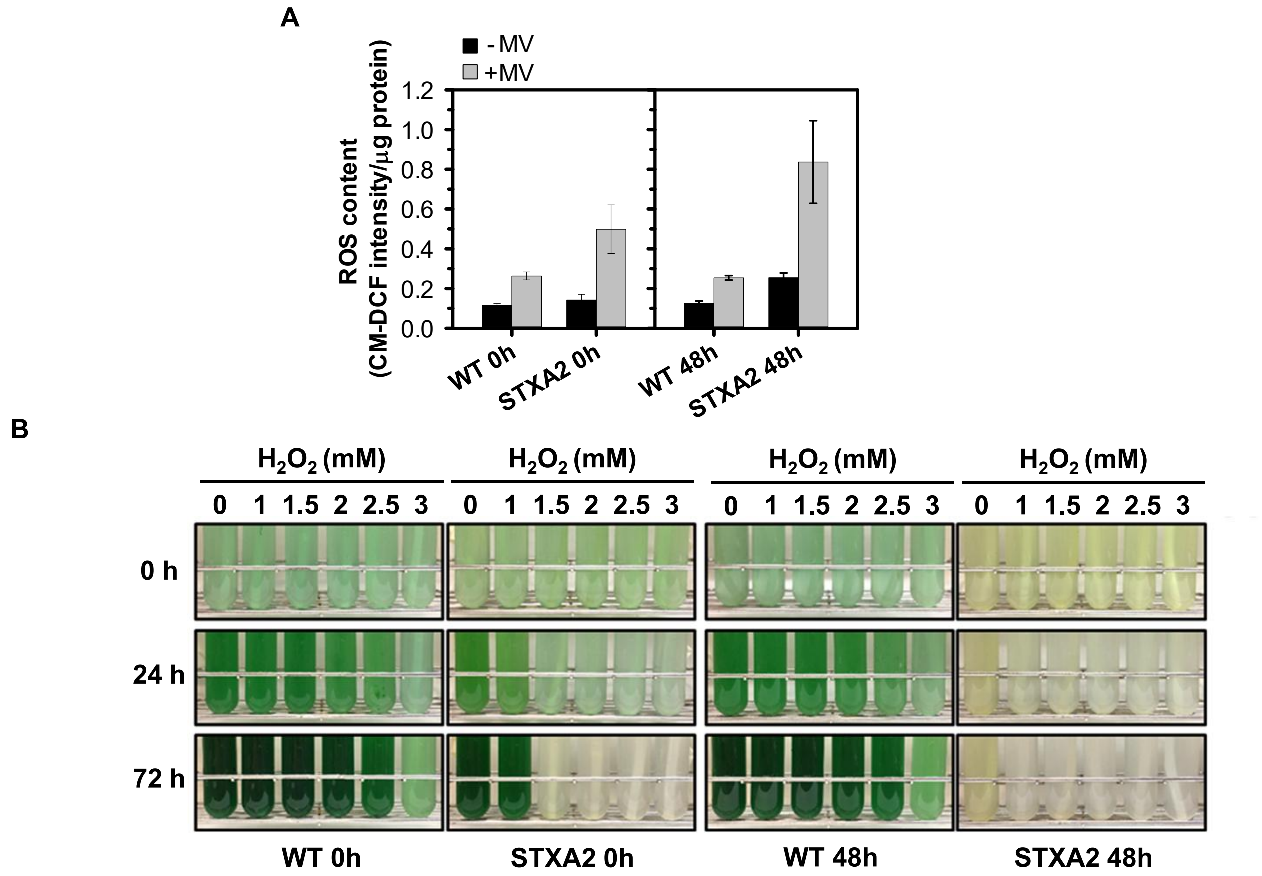

Supplemental Figure S8. Response to methyl viologen (MV) and hydrogen peroxide (H_2O_2) in WT and STXA2 strains. (A) Relative amounts of ROS in WT and STXA2 before (0h) and after 48 hours of inducer removal treated with 5 µM MV for 1 hour using the CM-H_2DCFDA reagent. Data are means ± SD from three biological replicates in all cases. (B) Effect of H_2O_2 on WT and STXA2 survival. Cells were collected before (0h) and after 48 hours of inducer removal (48h), diluted to a concentration of $2 \cdot 10^7$ cells·ml and incubated for 72h under standard conditions in the presence of up 3 mM H_2O_2.

Supplemental Table S1. Oligonucleotides used in this work.

Name	Sequence	
sll0586_F1	CAAAGCAATGGTGGGTCACATTAC	For pGQ5.4+ plasmid construction
TrxA_R1	GTCGGATCCTTGAGGGGTAGCACTCAT ACTG	For pGQ5.4+ plasmid construction
sll0585_F1	GCTACCCCTCAAGGATCCACCCCCAAC	For pGQ5.4+ plasmid construction
sll0585_R1	CCATGGAACGCTATGGTCTTACCGAC	For pGQ5.4+ plasmid construction
TrxA_NdeI	AATTCATATGAGTGCTACCCCTCAAG	For pBS-TRXA2 plasmid construction
TrxA_NotI	AATTGCGGCCGCCACAAAGCAGGGAA GCCAG	For pBS-TRXA2 plasmid construction
TrxA_1 (3)	TAGGAGCGCCATATGAGTGCTACCCCT CAAGTTTCC	For STXA2 segregation
TrxA_2 (4)	AAACCCCGGGAAGTGATTGGCGAATT AG	For STXA2 segregation
glnN_check_F (50)	ATGCAGGCCAGTCTTCCTAA	For STXA2 segregation
glnN_check_R (51)	AAATGGCAGTGTCCAAGTCC	For STXA2 segregation
Prom_Ck1_R (41)	AAGAGTGGTACCCATGGTAAACGATCC TCATCCTGTCTC	For STXA2 segregation
rnpB_F	GAGTTGCGGATTCCTGTCAC	*rnpB* probe
rnpB_R	AATTCCTCAAGCGGTTCCAC	*rnpB* probe
trxA1	TTCCAGTATGAGTGCTACCC	*trxA* probe
trxA2	AAGCCAGCGCTTAAAGATAT	*trxA* probe

Supplemental Table S2. Antibodies used in this work.

Name	Source	Titer
αTrxA	Lab collection	1:3000
αTrxB	Lab collection	1:2000
αTrxQ	Lab collection	1:2000
αGrxA	Lab collection	1:1000
αGrxC	Lab collection	1:3000
αFTR	Lab collection	1:5000
αD1	Agrisera (AS11 1786)	1:10000
αCP47	Agrisera (AS04 038)	1:3000
αPsaB	Agrisera (AS10 695)	1:5000
αPsaD	Gaozhong Shen (Pennsylvania State University)	1:7000
αAtpB	Agrisera (AS05 085)	1:5000
αPetA	Agrisera (AS06 119)	1:10000
αChlI	C. Neil Hunter (University of Sheffield)	1:5000
αPC	Raúl V. Durán (University of Seville)	1:10000
αFNR	Ghada Ajlani (CNRS)	1:10000
α2-Cys Prx	Lab collection	1:5000
αFBP/SBPase	Lab collection	1:10000
αRbcL	Agrisera (AS03 037)	1:10000
